# Altered lipid metabolism in the aging kidney identified by three layered omic analysis

**DOI:** 10.18632/aging.100900

**Published:** 2016-02-16

**Authors:** Fabian Braun, Markus M. Rinschen, Valerie Bartels, Peter Frommolt, Bianca Habermann, Jan H.J. Hoeijmakers, Björn Schumacher, Martijn E.T. Dollé, Roman-Ulrich Müller, Thomas Benzing, Bernhard Schermer, Christine E. Kurschat

**Affiliations:** ^1^ Department II of Internal Medicine and Center for Molecular Medicine Cologne, University of Cologne, Cologne, Germany; ^2^ Cologne Excellence Cluster on Cellular Stress Responses in Aging-associated Diseases (CECAD), University of Cologne, Cologne, Germany; ^3^ Department of Cardiology and Angiology, University of Münster, Münster, Germany; ^4^ Systems Biology of Ageing Cologne, University of Cologne, Cologne, Germany; ^5^ Max Planck Institute of Biochemistry, Martinsried, Germany; ^6^ Department of Cell Biology and Genetics, Medical Genetics Centre, Erasmus MC, University Medical Centre Rotterdam, Rotterdam, The Netherlands; ^7^ Institute for Genome Stability in Aging and Disease, Medical Faculty, University of Cologne, Cologne, Germany; ^8^ National Institute of Public Health and the Environment, Centre for Health Protection, Bilthoven, The Netherlands

**Keywords:** renal aging, gene expression profiling, microarray analysis, lipidomics, proteomics

## Abstract

Aging-associated diseases and their comorbidities affect the life of a constantly growing proportion of the population in developed countries. At the center of these comorbidities are changes of kidney structure and function as age-related chronic kidney disease predisposes to the development of cardiovascular diseases such as stroke, myocardial infarction or heart failure. To detect molecular mechanisms involved in kidney aging, we analyzed gene expression profiles of kidneys from adult and aged wild-type mice by transcriptomic, proteomic and targeted lipidomic methodologies. Interestingly, transcriptome and proteome analyses revealed differential expression of genes primarily involved in lipid metabolism and immune response. Additional lipidomic analyses uncovered significant age-related differences in the total amount of phosphatidylethanolamines, phosphatidylcholines and sphingomyelins as well as in subspecies of phosphatidylserines and ceramides with age. By integration of these datasets we identified Aldh1a1, a key enzyme in vitamin A metabolism specifically expressed in the medullary ascending limb, as one of the most prominent upregulated proteins in old kidneys. Moreover, ceramidase Asah1 was highly expressed in aged kidneys, consistent with a decrease in ceramide C16. In summary, our data suggest that changes in lipid metabolism are involved in the process of kidney aging and in the development of chronic kidney disease.

## INTRODUCTION

Developed countries face an enormous increase in the elderly population. Estimates predict an increase in life expectancy to 88 years for men and to 91 years for women aged 65 in 2030 [1]. With this change in demo-graphics comes the necessity to better understand the mechanisms of aging in different organs and associated diseases. Chronic kidney disease (CKD), as an example, has already become a major health and economic burden that will further increase in the future [1-4]. Renal aging involves cellular and structural changes within the kidney and has important implications for aging-associated comorbidities especially cardio-vascular disease. On the molecular level an increase of oxidative damage and its products as well as an increase in cyclin-dependent kinase (CDK) inhibitors such as p16, has been reported [2], leading to senescence especially in the cortical tubular system [4,5]. This contributes to a chronic inflammatory response with accumulating macrophages and lymphocytes in the interstitium [1-4]. Histopathological consequences are tubular damage with tubular atrophy and interstitial fibrosis. In addition, glomerulosclerosis is a hallmark of age-related kidney disease [1,2,4]. Macroscopically, aged kidneys develop cysts and a global loss in weight and mass while, functionally, these alterations lead to potassium retention and impairment of sodium and fluid balance. In addition, the response of erythropoietin to anemia and also vitamin D activation are reduced with age. These developments are worsened with underlying diseases such as diabetic nephropathy or cardiovascular disease [1,2,4,5]. Vice versa, CKD as a major contributor and risk factor aggravates cardiovascular disease [2,6]. However, the exact underlying molecular mechanisms for these changes remain largely unknown.

The aim of this study was to gain a better understanding of the mechanisms underlying renal aging by employing a three-layered omic strategy. We characterized age-related transcriptional changes comparing gene expression in kidneys of young and aged wild-type mice. Our results identified genes involved in lipid metabolism to be differentially expressed in aged kidneys. These findings were partially confirmed on the protein level. They are consistent with changes in lipidomic profiles suggesting dysregulated lipid metabolism as a pathogenic factor and potential target of future therapeutic strategies.

## RESULTS

### Mice develop a distinct kidney aging phenotype with up-regulation of kidney damage markers

**Figure 1:**
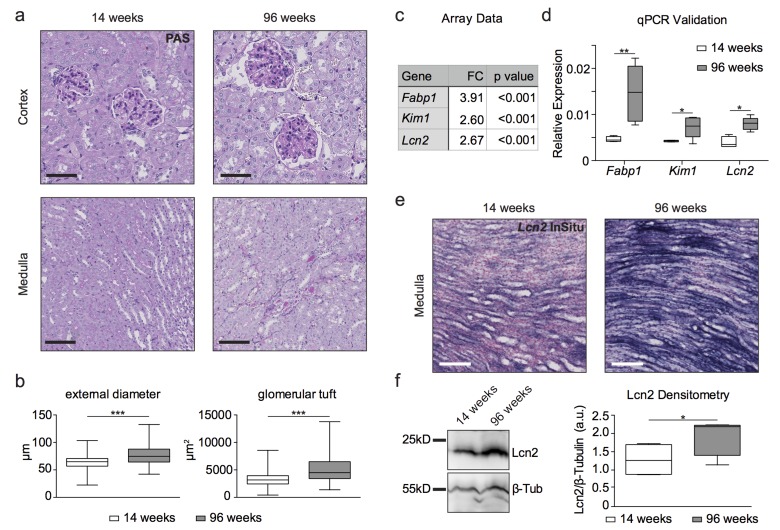
Histology, transcriptome analysis, western blot and in situ hybridization reveal a kidney aging phenotype **(a)** PAS staining of young and aged wildtype kidneys. Aged kidneys show cysts and hypertrophic glomeruli, prominent basal membranes and dilated capillary loops in the renal cortex as well as protein cylinders in parts of the medulla. Scale bars - upper panel: 50μm; lower panel: 100μm **(b)** Quantitative measurement of glomeruli by their external diameter and glomerular tuft area. Aged glomeruli show a hypertrophy compared to 14 week old glomeruli. **(c)** Table of fold change (FC) in kidney damage markers obtained from microarray analysis. **(d)** qPCR validation of array data for kidney damage markers. **(e)** In situ hybridization for Lcn2 (NGAL)-RNA on formalin-fixed paraffin-embedded kidney tissue. 96 week old kidneys show increased Lcn2 RNA levels in the papilla compared to young animals. Scale bar: 100μm **(f)** Immunoblot for Lcn2 shows a clear increase in protein content in 96 week old kidney lysates. β-tubulin was used as a loading control and for normalization of densitometry. Boxplots depict mean values with whiskers showing 5-95% percentile.*p<0.05, **p<0.01, ***p<0,001.

Histological examination of 96 week old wild type (wt) C57BL6 mouse kidney tissue revealed renal cysts, dilated tubules and hypertrophic glomeruli as well as prominent basal membranes and dilated capillary loops in the renal cortex compared to 14 week old renal tissue (Fig. 1a). Aged kidneys also displayed protein cylinders in parts of the medulla. Analysis of glomerular size and diameter showed glomerular hypertrophy with increased glomerular tuft area and increased external diameter in aged glomeruli (Fig. 1b). We detected an increase of kidney damage markers such as fatty acid binding protein 1 (Fabp1), kidney injury molecule-1/hepatitis A virus cellular receptor 1, (Kim1/Havcr1) and neutrophil gelatinase-associated lipocalin*/*lipocalin 2 (Ngal/Lcn2) in old kidneys on the transcriptomic level (Fig. 1c). These data were confirmed by qPCR (Fig. 1d). Increased Ngal/Lcn2 expression occurred primarily in the kidney medulla as shown by in situ hybridization (Fig. 1e) and could be detected on the protein level in immunoblots of whole kidney lysates (Fig. 1f).

### Genes involved in lipid metabolism are differentially expressed in old wt kidneys

Using microarray analysis of total RNA from 14 and 96 week old kidney tissue we detected 25.528 annotated genes (www.ncbi.nlm.nih.gov/geo/query/acc.cgi?acc=GSM1921101).

**Figure 2:**
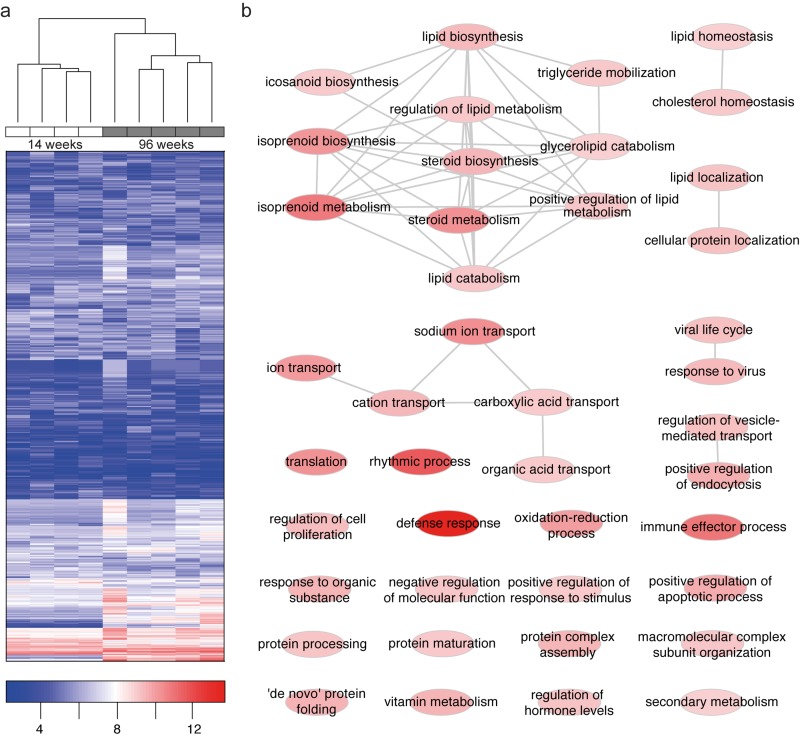
Microarray analysis reveals age-specific clustering and differentially expressed genes to be associated with lipid metabolism **(a)** Hierarchical clustering of 1000 genes with the strongest variation reveals age-specific clustering. Genes with low expression are depicted in blue color, genes with high expression in red color. **(b)** Gene ontology (GO) enrichment of differentially expressed genes (96W – 14W WT kidney). We observed a strong enrichment of lipid and lipoprotein metabolism, of immune system and defense response, of small ion transport and transmembrane transport in aged wild-type kidneys (see also Data Set 2). Nodes of GO terms are color-coded according to enrichment strength.

Of these genes 420 were found to be differentially expressed between age groups with a clear age-specific clustering (Fig. 2a). Fold changes were validated by qPCR analysis of 11 candidate genes (Fig. S1). We performed gene ontology (GO) analyses to better understand common pathophysiologic mechanisms. A substantial number of genes differentially expressed in young versus old kidney tissue are related to lipid metabolism. In particular, genes involved in modifying sterol and lipid metabolism represent the most prominent cellular processes influenced by aging (Fig. 2b,c,). Additionally, differentially expressed genes were involved in cellular and immune responses in both GO and network analysis (Fig. 2b, Fig. S2).

### Proteomic analysis reveals alterations in proteins involved in lipid metabolism

We analyzed lysates of 14 week old kidneys and 96 week old kidneys by label-free quantitative mass spectrometry. More than 2000 proteins were detected and 1300 high-confidence proteins were quantified (Data Set 1). To indicate which protein functions were affected, we performed GO term analysis of the changed proteins as compared to the non-changed proteins. Among the 65 changed proteins, the most significantly enriched GO term was “cellular lipid metabolism” (−log (p) = 2.65), followed by “cellular amino acid metabolic process (−log (p) = 2.59) and lipid transport” (−log (p) = 2.48).

### Integration of proteomic and transcriptomic data

**Figure 3:**
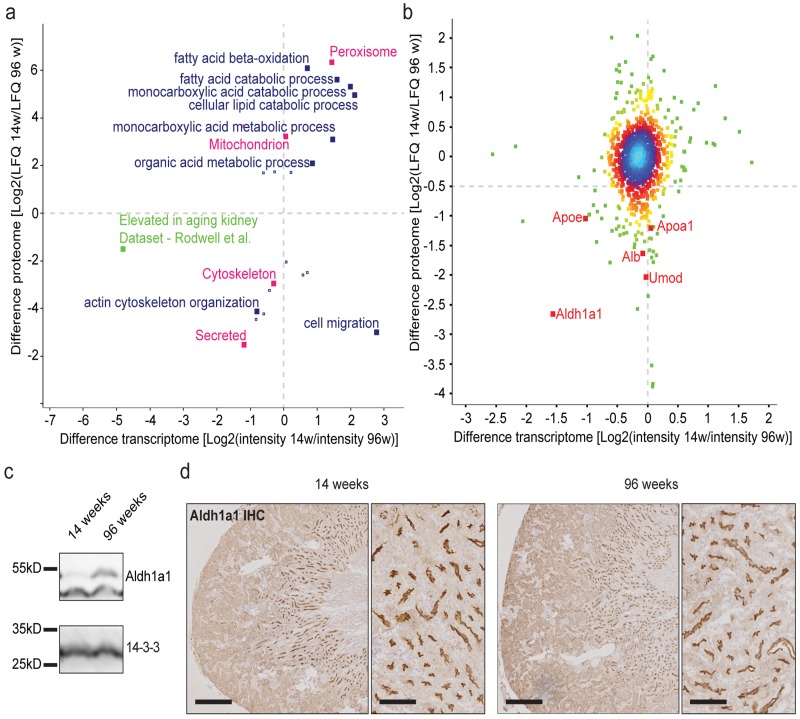
Proteome analysis depicts little correlation between transcriptome and proteome. Aldh1a1 is identified as an aging marker in the kidney **(a)** 2D GO enrichment analysis. Positive values indicate higher RNA expression or protein abundance per GO term in 14 week old samples. Negative values show higher expression / abundance per GO term in 96 week old samples. Blue: Gene ontology biological process (GOBP), red: selected UniProt keywords, green: representation of published geneset **(b)** Correlation analysis of transcriptome and proteome differences between age groups. Positive values indicate higher RNA expression or protein abundance in 14 week old samples. Negative values show higher expression / abundance in 96 week old samples. Colors depict proximity values ranging from blue (very close together) to dark green. Aldh1a1 shows the highest difference and correlation. **(c)** Immunoblot for Aldh1a1 shows a clear increase in protein content in 96 week old kidney lysates. 14-3-3 was used as a loading control. (d) Immunohistochemistry for Aldh1a1 on formalin-fixed paraffin-embedded mouse kidney tissue. Staining showed a clear localization to the brush border of the medullary thick ascending limb (mTAL) segment. This staining did not vary in intensity and localization between age groups. **p<0.01; Scale bars in left panels indicate 400μm. Scale bars in right panels indicate 100μm.

We correlated fold-changes detected by mass spectrometry analyses of 14 and 96 week old whole kidney lysates to all differentially expressed genes annotated in our microarray analysis (Data Set 1). After annotation to Gene ontology biologic processes (GOPBs), UniProt keywords and published gene sets, we performed a 2D GO enrichment analysis [7-9] (Fig. 3a). GOBPs for lipid metabolism are differentially regulated on both transcriptomic and lipidomic level. The published gene set for aged human kidneys by Rodwell et al. is also reflected by our mouse kidney data [2,5,7,10,11]. We observed a weak but significant correlation between mRNA transcription and corresponding protein abundance (r=0.111; p<0.0001, Fig. 3a), indicating that transcription may influence aging-associated differences in cellular protein abundance, although the effect of transcription appears to be small.

Mapping transcriptome and proteome data, aldehyde dehydrogenase family 1 subfamily A1 (Aldh1a1) exhibited the strongest correlation, with an age-related fold-change of 1.56 (on a log2 scale) on the transcriptome and a fold-change of 2.66 (on a log2 scale) on the proteome level (Fig. 3b). We validated this finding by Aldh1a1 immunoblot of whole kidney lysates from young and old mice showing an increase in Aldh1a1 protein abundance in aged kidneys (Fig. 3c). Immunohistochemistry (IHC) for Aldh1a1 identified the medullary part of the thick ascending limb of the loop of Henle as the main segment for its expression (Fig. 3d). Interestingly, we detected an accumulation of apolipoprotein A and E (ApoA and ApoE) as well as uromodulin (Umod) on the proteome level with only little differential expression on the transcriptome level (Fig. 3b).

### Phospholipid mass spectrometry analysis shows differences in lipid species and subspecies between young and aged mouse kidneys

**Figure 4:**
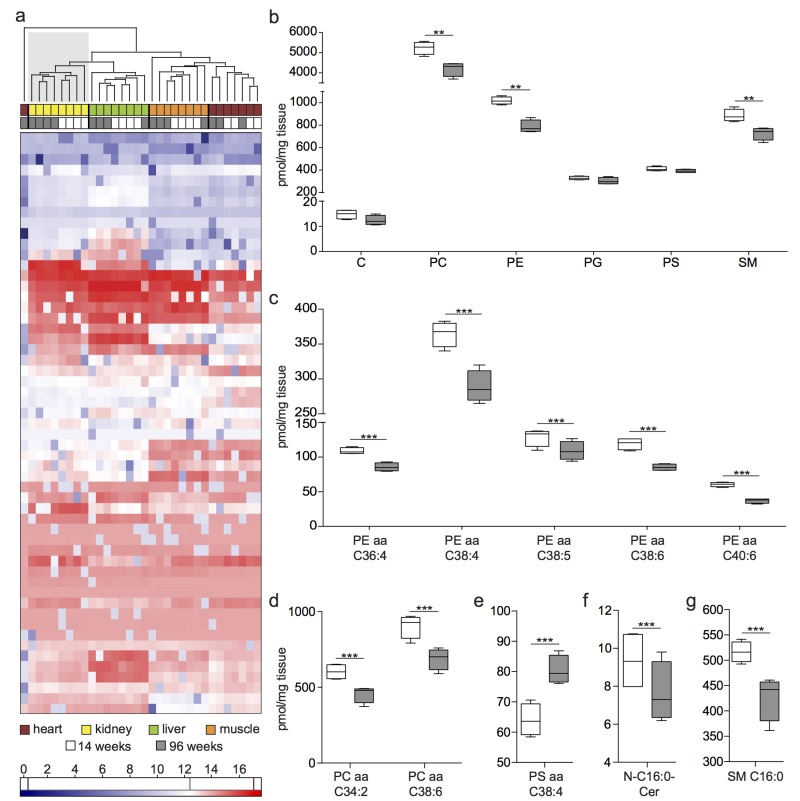
Lipidome analysis shows age-specific clustering and a decrease of lipid species and subspecies with age **(a)** Hierarchical clustering of lipid subspecies detected in all organ samples. Low abundant lipids are depicted in blue, high abundant lipids in red color. **(b)** Overall lipid sums detected in whole kidney lysates. C: Ceramide, PC: Phosphatidylcholine, PE: Phosphatidylehtanolamine, PG: Phosphatidylglycerol, PS: Phosphatidylserine, SM: Sphingomyeline **(c)** PE subspecies with significantly different abundance between age groups. **(d)** PC subspecies with significantly different abundance between age groups. **(e)** PS subspecies with significantly different abundance between age groups. **(f)** Cer subspecies with significantly different abundance between age groups. **(g)** SM subspecies with significantly different abundance between age groups. Boxplots depict mean values with whiskers showing 5-95% percentile. **p<0.01; ***p<0.001.

To integrate the proteomic and transcriptomic analysis into a broad analysis of lipid metabolism, we performed targeted mass spectrometry-based lipidomic analysis. Lipidomic analysis comparing young versus old kidney lysates detected an overall of 189 lipid subspecies in all of our samples (Data Set 2). We performed hierarchical clustering analysis of the absolute lipid concentrations and found an age-specific clustering for all aged kidney samples and all young kidney samples (Fig. 4a). Interestingly, this age-related separation of samples was not detected in liver, heart and muscle samples. When looking at the overall content of phosphatidylcholine (PC), phosphatidylethanolamine (PE), phosphatidyl-glycerol (PG), phosphatidylserine (PS), ceramides (Cer) and sphingomyelins (SM), we detected a significant decrease for PC, PE and SM in aged kidneys (Fig. 4b).

With traditional biochemical staining methods based on PE or SM binding agents we did not detect a difference between 14 and 96 week old kidney tissue in immunofluorescence (Fig. S3). None of the other organs investigated (liver, heart, muscle) revealed a lipid pattern similar to the kidney pattern. Since both PE and PC were detected at lower levels in 96 week old kidneys, we examined the PE:PC ratio. We found no significant difference between age groups (Fig. S3). We detected a significant decrease in lipid subspecies depending on their overall fatty acid chain length and number of double bonds. Data for significant differences in abundance of PE, PC, PS, Cer and SM are shown in Fig. 4c, d, f. PS aa C38:4 was significantly more abundant and N-C16:0-Cer significantly less abundant in aged kidneys (Fig. 4e, g).

### Asah1 shows a higher abundance in aged kidneys and correlates with a decrease in bioactive ceramide C16

**Figure 5:**
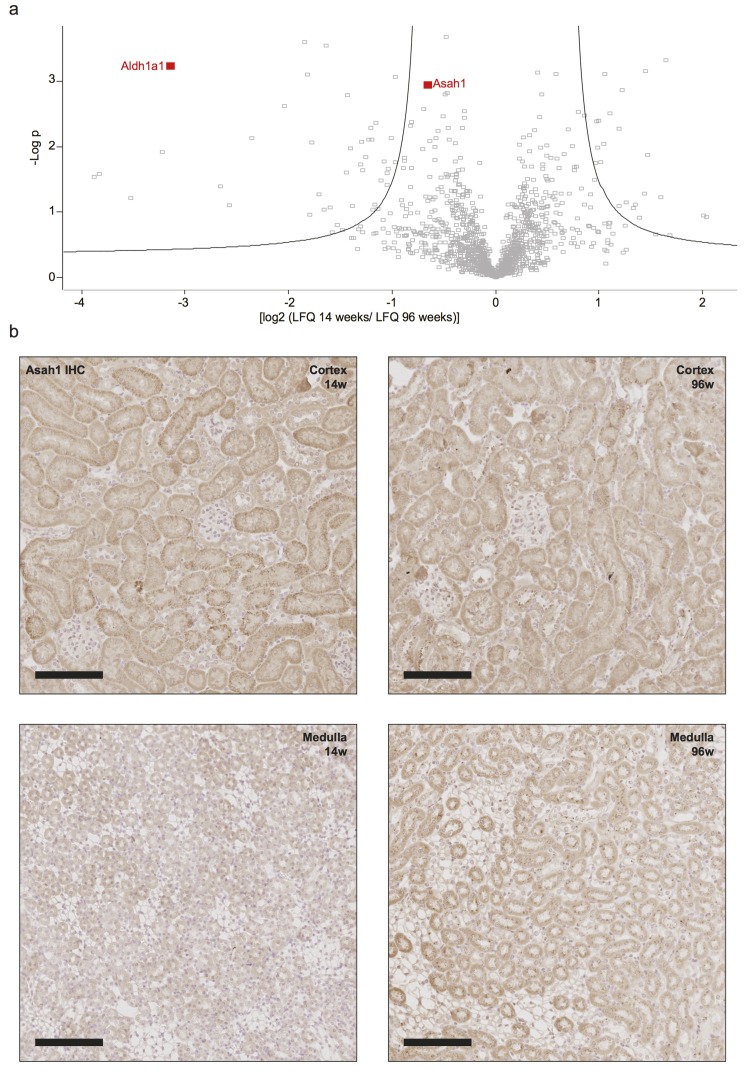
Increased abundance of Asah1 correlates with a decrease in ceramide 16 in aged kidneys **(a)** Volcano plot of differences in protein abundance between age groups. Positive values indicate higher protein abundance in 14 week old samples. Negative values indicate higher abundance in 96 week old samples. Asah1 was detected to have a fold change of 0.69 in aged kidneys. Aldh1a1 is highlighted as a comparison. **(b)** Immunohistochemistry for Asah1 on formalin-fixed, paraffin-embedded mouse kidney tissue showing low expression in the cortical tubular system with no expression in glomeruli and decreased expression in the young medulla. Scale bars indicate 100μm.

Since important classes of lipids were significantly altered within the aging kidney, we re-examined the data obtained from proteomics to identify corresponding regulations on the proteome level. We mapped each metabolite to corresponding proteins involved in its synthesis or degradation (see methods for details, Data Set 3). Two metabolites were related to the detected proteins in our proteome analysis, with N-acyl-sphingosine amidohydrolase 1 (Asah1) showing a significantly higher abundance in aged kidneys (Fig. 5a). Asah1 is a ceramidase catalyzing the degradation of ceramides which nicely correlates to the decrease we see in N-C16:0-Cer (Fig. 4f). Immunohistochemistry for Asah1 identified the protein to be expressed mostly in the cortical tubular network with an increase in abundance in the medulla of aged kidneys (Fig. 5b).

## DISCUSSION

Gene expression profiling provides major insights into genetic mechanisms of the aging process. RNA expression profiles and proteomic analyses of different organs have identified age-related changes in different tissues, including the kidney [1,12-14]. Recently, the metabolome has revealed strong predictors of healthy aging, longevity as well as brain aging and neurodegenerative disease [15-18]. The metabolome and its subdiscipline, the lipidome, represent a functional readout of cellular biochemistry [19]. Thus, the integration of information on lipidomics with proteomics and expression profiles can help to shape the understanding of metabolic pathways as potential targets for future therapeutic interventions [20]. In this study, we used a three-layered multi-system approach to identify genes and pathways involved in kidney aging. Lipid metabolism was among the most prominent hallmarks of kidney aging [12,21,22]. A previous study investigating gene expression of liver, kidney and brain of 33 different mammalian species identified genes involved in lipid oxidation and lipid modification to be negatively correlated with lifespan and organism maturity time [23]. Comparing transcriptomic patterns of prematurely aged and long-lived mice, Schumacher et al. detected genes involved in lipid metabolism among genes differentially expressed during the aging process in various tissues including the kidney [24]. In agreement with data on RNA profiles of 3 and 23 month old C57BL6 mice we detected a decrease in acetyl coenzyme A oxidases on the protein level in our aged samples [25]. However, our data did not reflect higher levels of renal triglycerides and cholesterol or an increase in 3-hydroxy-3-methylglutaryl-coenzyme-A reductase (HMGCR) with aging, as previously described [25]. We detected a decrease in HMGCR mRNA, the key enzyme in cholesterol synthesis. These findings support the hypothesis that the aging process specifically affects the synthesis of lipids and their bioactive derivatives like sterols. Interestingly, carnitine palmitoyltransferase 1a (Cpt1a) protein abundance was decreased in the proteome of aged kidneys (Tbl.S1). This is in accordance with a previous study in which Cpt1a expression was inhibited in diabetic nephropathy, a common aging-associated disease [26].

The role of lipids in aging, disease and longevity has been described previously [25,27-29]. In our analysis, we focused on phospholipids known to be essential cellular components and signaling molecules. Lipidomic analyses exhibited age-specific alterations in phosphatidylcholine, phosphatidylethanolamine and sphingomyelin species and subspecies. Lipid composition in our study clearly distinguished old from young kidney samples (Fig. 4a) with a decrease in phospholipids with age. The correlation of this decrease with aging-related diseases is further supported by a study on the offspring of nonagenarians, revealing a significant increase in phosphatidylcholine and sphingomyelin plasma levels compared to controls to be associated with a lower risk for hypertension and diabetes [17]. Furthermore, a potential pathologic role for a decrease in PE and the subspecies PE C38:4, PE C38:5, PE C38:6 and PE C36:4 was recently hypothesized by a study comparing the lipid composition of kidney tumors to surrounding tissue [30]. However, the ratio of phosphatidylcholines (PC) versus phosphatidylethanolamines (PE), a parameter with importance for membrane integrity and progression to a high fat-induced disease [31], was not changed (Fig. S3a).

Of specific interest is a significant decrease in bioreactive ceramide within the aging kidney. This is consistent with an increase in RNA and protein abundance of the acid ceramidase Asah1 in old kidneys. Asah1 mediates the hydrolysis of ceramide into sphingosine and free fatty acids [32]. An increase in sphingolipid-degrading enzymes has been demonstrated in aging rat kidneys which is supported by our data [33]. Ceramides as bioactive lipids have been shown to modulate Ca^2+^-signaling in basolateral membranes of proximal tubular cells *in vitro* [34]. An increase in total plasma ceramide levels was detected in mice fed a high fat diet and corresponded with renal injury [35]. A decrease in ceramides, as observed in aged kidneys in this study, may lead to alterations in cellular survival, apoptosis as well as migration and cytoskeletal rearrangements [36-38]. This is in part mediated through the catabolic product of Asah1, sphingosine, and its phosphorylated form (S1P). As a ligand for five G protein-coupled receptors, S1P has already been shown to mediate renal fibrosis directly *in vitro* and *in vivo* [39]. Thus, alterations in ceramide metabolism may directly influence the aging process in the kidney.

Correlating transcriptome and proteome data in our study revealed a coefficient of 0.111. Compared to previous studies investigating transcriptome and proteome correlations in mice, zebrafish or human cells, this coefficient is rather low [40-42]. Nevertheless, numerous studies suggest that age-related changes may also include posttranslational modifications and alterations in protein degradation [43,44]. More importantly, our whole kidney proteome also reflects proteostatic changes associated with aging, which consist of protein aggregates and deposits also in the extracellular system. This is supported by an increased abundance of Tamm-Horsfall protein (Umod) and albumin (Alb) in the aging kidney correlating with loss of filter function and the formation of tubular protein casts (Fig. 1a, Fig. 3b). We identified Aldh1a1 to be increased on both the transcriptomic and proteomic level in aged kidneys. Aldh1a1 is a key enzyme in lipophilic vitamin A metabolism, converting retinal to retinoate. Interestingly, Aldh1a1 expression is selectively regulated by sterol regulatory element-binding protein (SREBP-1c), a transcription factor involved in regulation of sterol metabolism-associated genes [45]. Target genes include ATP citrate lyase, acetyl-CoA carboxylase and fatty acid synthase [46,47]. It has been demonstrated that SREBP-1c expression is increased in aging mouse kidneys [25] as well as in kidneys of a diabetic mouse model with a subsequent decrease in all-trans-retinoic acid and Pparβ/δ [48]. This supports the hypothesis that Aldh1a1 and its regulation play a prominent role in the kidney aging process, but mainly in the thick ascending limb of the loop of Henle (Fig. 3b).

Beyond lipid metabolism, our data display changes in biomarkers of kidney disease. Consistent with the transcriptomic data of a study on young and aged human kidneys we found apolipoprotein E and Fabp1, a potential marker of acute kidney injury, to be increased in aged kidney samples pointing to a conserved aging mechanism [49, 50]. Two additional kidney damage markers, Kim1 and Ngal/Lcn2, were found to be upregulated on the transcriptional level in aged kidneys in our study (Fig. 1). These markers have been investigated in numerous studies as potential new biomarkers for acute kidney injury in human [50-52]. In concordance with a previous analysis, our study suggests that these proteins could also be useful markers of renal damage during kidney aging, and their role during the aging process needs further characterization [53].

We demonstrate that lipid metabolism is altered on both the transcriptomic and the proteomic level with clear, potential decisive links towards a dominant aging-associated lipid phenotype. In particular, we show that sphingolipid signaling and the vitamin A pathway play an important role in the aging process. The three-layered omics strategy exemplified here can be useful to discover, understand and prioritize untapped molecular pathways in kidney aging and disease.

## MATERIALS AND METHODS

### Mice

Mice were bred in either C57BL6 (proteomic, lipidomic, in situ hybridization) or mixed FVB/C57BL6 (microarray analysis & histology) background. All experiments were conducted according to institutional and federal guidelines and approved by the IACUC in Bilthoven, NIH/NIA 1PO1 AG 17242. Following federal regulations, the Animal Care Committee of the University of Cologne reviewed and approved the experimental protocol. Animals were housed at specific pathogen-free (SPF) conditions with three-monthly monitoring according to FELASA suggestions. Housing was done in groups of less than six adult animals receiving CRM pelleted breeder and maintenance diet irradiated with 25 kGy (Special Diet Services, Witham, UK), and water *ad libitum*.

For RNA preparation and Affymetrix array analysis 5 mice were sacrificed at 14 and 96 weeks of age. 4 animals of 14 and 96 weeks of age were sacrificed for lipidomic and proteomic analysis.

Renal tissue was embedded in OCT (Sakura, Torrance, CA) and frozen at −80°C or fixed in 4% neutral buffered formalin and subsequently embedded in paraffin.

### Preparation of RNA

Mice were anaesthetized by intraperitoneal injection of 10 μl per g bodyweight of 0,01% xylocaine and 12,5 mg/ml ketamine – and perfused with cold phosphate buffered saline (PBS). Kidneys were excised and snap frozen in liquid nitrogen. Total RNA was extracted and purified using commercial homogenization (Bio 101 FastPrep FP120-120 V, Savant, Midland, MI, USA) and the RNeasy kit (Qiagen, Hilden, Germany).

### Microarray hybridization

Reverse transcription of RNA was done using the Applause WT-Amp ST RNA Amplification System (NuGen Technologies, Inc., San Carlos, CA, USA) in accordance to the manufacturer's protocol. cDNA probes were labelled with Encore Biotin Module (NuGen Technologies, Inc.), hybridized to the Affymetrix GeneChip Mouse Gene 1.0 ST Array according to the manufacturer's instructions and scanned with a GeneChip 3,000 6G scanner.

### Affymetrix microarray data analyses

Raw data (CEL files) were processed using the robust multi-array average (RMA) algorithm and quantile normalization with the Affymetrix Power Tools, version 1.12.0, and platform-specific library files [54]. Differential gene expression was analyzed using descriptive statistics (fold change) and Student's T-Test method for pairwise comparisons. Genes were prioritized by statistical evidence. In order to create candidate lists for differential gene expression between conditions, we used all genes regulated at least 1.5-fold where differential expression was significant at level 0.05. Type I error inflation was ignored because the p-values were used to prioritize the list rather than being interpreted in a confirmatory sense.

All microarray data reported in this study are described in accordance with MIAME guidelines and have been deposited in the National Center for Biotechnology Information Gene Expression Omnibus (GEO, http://www.ncbi.nlm.nih.gov/geo/) public repository. The data sets supporting the results of this article are available in the GEO public repository at: www.ncbi.nlm.nih.gov/geo/query/acc.cgi?acc=GSM1921101.

### Quantitative RT-PCR

For control purposes we assessed RNA expression of high-scoring genes from our microarray analyses using quantitative real-time PCR. After cDNA amplification we used SYBR green on an ABI 7900 HT thermocycler (Applied Biosystems, Life Technologies Cooperation, Carlsbad, CA, USA). Expression levels of housekeeping genes HPRT and ACTB were employed for normalization, and RNA expression levels were calculated with the comparative threshold cycle (Ct) method as previously described [55]. Primer sequences are provided in Supplemental Table S1.

### Annotation and enrichment analysis

DAVID [56] was used for gene annotation, as well as enrichment analysis. To visualize GO enrichments using REVIGO [57], we first translated GO-terms to GOslim using GO:TermFinder [58]. Visualization for Fig. 2 was done using Cytoscape [59].

Pathway enrichment analysis was done using the Reactome FI plugin [60] for Cytoscape. One linker was allowed. Visual preparation of the network file was done in Cytoscape and Illustrator^**™**^.

### Lipidomic analysis

Snap frozen kidneys, hearts, brains and skeletal muscles were sent to Biocrates Inc., Innsbruck, for lipid mass spectrometry. The biologically most abundant members of (lyso-) glycerophospholipids, i.e. (lyso-) glycerophospho-cholines, -ethanolamines, -serines, -glycerols, and sphingolipids, i.e. sphingomyelins, ceramides, dihydroceramides, and 2-hydroxyacyl ceramides were quantitatively analyzed by a high throughput flow injection ESI-MS/MS screening method. The MRM detection in positive and negative mode was performed using a 4000 QTrap® tandem mass spectrometry instrument (AB SCIEX). The sample preparation of 20μL sample volume followed a MeOH/CHCl3 -liquid/liquid-extraction protocol. Besides five internal standards to compensate for matrix effects, 43 external standards were used for a multipoint calibration. The quantitative data analysis was performed with Biocrates MetIDQ™ enabling isotopic correction and basic statistical analysis.

Clustering analysis was done using Perseus software with euclidian distances. JMP (SAS, Böblingen) was used for quantitative analysis (3way-ANOVA) and visualization of results.

### Proteomic analysis

Snap frozen kidneys were thawed in 8M urea buffer and complete protease inhibitors (PIM, Roche) and dounce homogenized on ice 20 times. Suspension was sonicated at 10% amplitude, 10% pulse for 2 seconds and centrifuged at 4°C full speed for 15 minutes. Supernatant was purified using C18 StageTips as previously described [61].

### LC-MS/MS and search parameters

Samples were analysed using a nLC (90 min gradient separation on a 15m C18 column (parameters in [62]) coupled to a QExactive plus machine (Thermo Scientific) with machine settings as previously described [63]. Raw files were searched using MQ v 1.4.1.2 against a recent mouse reference proteome database (uniprot) including a list of contaminants [64] using default parameters. Decoy mode was revert. PSM, protein and site FDR was set to 0.01. Minimum peptide length was 7AA. Fixed modification was carbamidomethylation on cysteins. Variable modifications included in protein quantification were acetyl (Protein N-term) and methionine oxidation. Label-free quantification and match between runs option was enabled.

### Bioinformatic analysis

Protein groups file was imported in Perseus (v 1.5.0.24) [8] and processed as previously described with few modifications [65]. Common contaminants, reverse hits and proteins identified by site only were entirely removed from the dataset. Logarithmized intensities were normalized and missing values (at least 3 valid values in at least 1 group had to be present) were imputed according to the normal distribution (downshift 1.8 SD, width =0.3). LFQ of 14 and 96 week old kidney samples were compared using a two-tailed t-test with correction for FDR by using a method similar to SAM [66]. Cutoff was s0=0.1 and FDR was set to less than 0.2. Proteins were annotated with GO terms, KEGG pathways, and gene sets using the Perseus annotation package. Normalized expression values from microarray analysis were imported and logarithmized. Data were merged based on the gene symbol matches. 2D enrichment of annotations based on the differences [log2(signal 14 weeks/signal 96 weeks)] of the transcriptomic and proteomic dataset was performed [8]. The permutation based FDR cutoff was set to 0.05.

### Western blot

The snap frozen kidney samples were minced and lysed in 1% Triton X-100 buffer [1% Triton X-100, 20 mM Tris·HCl pH 7.5, 50 mM NaCl, 50 mM NaF, 15 mM Na4P2O7, 2 mM Na3VO4, and complete protease inhibitors (PIM; Roche)] by dounce homogenization and sonication at 10% amplitude, 10% pulse for 1m20s on ice. After centrifugation at 15,000 *g* for 15 min at 4°C protein content was measured using BCA assay (Life Technologies, Darmstadt). Supernatant was diluted according to protein mass with 1% triton X-100 buffer and 4% SDS sample buffer was added accordingly.

Size separation was done using SDS-PAGE. Samples were blotted onto polyvinylidene difluoride membranes and visualized with enhanced chemiluminescence after incubation of blots with corresponding antibodies (goat anti mouse lipocalin-2/NGAL antibody; R&D Systems, Minneapolis; mouse anti beta tubulin antibody; HybridomaBank).

### Immunohistochemistry (IHC) methods

Paraffin-embedded sections were deparaffinized in Xylene (VWR, Darmstadt, Germany) and rehydrated in decreasing concentrations of ethanol. Heat-induced antigen retrieval was performed in 10mM Tris 1mM EDTA 0,005% Tween buffer, pH9.0 for 15. Peroxidase blocking was performed in 3% hydrogen peroxidase (Roth, Karlsruhe, Germany). After incubation in primary antibody (anti-Aldh1a1 antibody, ab52492; anti-Asah1 antibody, ab74469 Abcam, Cambridge, UK) 1:200 in TBS 1% BSA at 4°C overnight, sections were washed in TBS and incubated in biotinylated mouse anti-rabbit secondary antibody (Jackson Immuno-research, West Grove, USA) 1h at room temperature. For signal amplification the ABC Kit (Vector, Burlingame, CA, USA) was used before applying 3,30-diaminobenzamidine (Sigma-Aldrich, St Louis, USA) as a chromogen. Hematoxylin was used for counter-staining. After dehydration slides were covered in Histomount (National Diagnostics, Atlanta, USA).

For the assessment of age-related histologic alterations periodic acid Schiff staining was used.

### Lipid staining

OCT-embedded sections were dried for 30 minutes at room temperature. After fixation in 4% PFA (Sigma-Aldrich) sections were washed in Dulbecco's PBS (DPBS) before blocking and permeabilization in 0,1% Triton, 2% BSA in DPBS for 1h at room temperature. Afterwards sections were washed again in DPBS.

For SM staining, sections were incubated in lysenin (PeptaNova, Sandhausen, Germany) 2μg/ml in DPBS at 4°C o/n, washed and reincubated in rabbit anti-lysenin serum (PeptaNova) in 2% BSA in DPBS at 4°C o/n. After washing in DPBS, sections were incubated in Cy3-coupled donkey anti-rabbit antibody (Jackson) 1:500 in 2% BSA in DPBS at room temperature for 1h, washed again and mounted in Prolong Gold + DAPI (Life Technologies).

For PE staining, sections were incubated in duramycin-LC-biotin (Molecular Targeting Technologies Inc., West Chester, USA) 0,5μg/ml in 2% BSA in DPBS at 4°C o/n. After washing, sections were incubated in Cy3-coupled streptavidin (Life Technologies) 1:2000 in 2% BSA in DPBS at room temperature for 1h, washed again and mounted in Prolong Gold + DAPI (Life Technologies).

### In situ hybridization

10 μm sections of paraffin-embedded kidney tissue were deparaffinised in xylene and rehydrated in decreasing concentrations of ethanol. In situ hybridization was carried out according to the GUDMAP protocol accessible at www.gudmap.org/Research/Protocols/McMahon/SISH.pdf”

## SUPPLEMENTAL DATA FIGURES, TABLE AND DATASETS








