# Supplementary material for: Altered lipid metabolism in the aging kidney identified by three layered omic analysis
Source: Aging (Albany NY). 2016 Feb 16;8(3):441–54. doi: 10.18632/aging.100900 (PMC4833139; doi:10.18632/aging.100900)
Supplement: Supplementary file 1 [file aging-08-441-s001.pdf]

SUPPLEMENTAL DATA

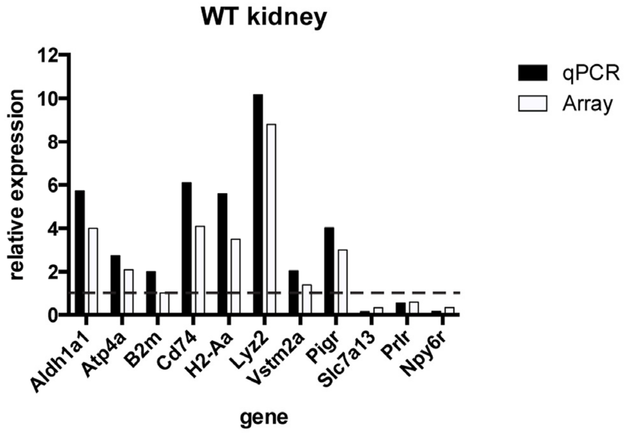

Figure S1. qPCR validation of array data. The regulation of 10 randomly selected genes from the microarray analysis was validated by using quantitative real-time PCR. All genes investigated were regulated in the same direction in microarray datasets and in qRT-PCR. Expression levels of housekeeping gene B2M as set to 1 (dotted line).

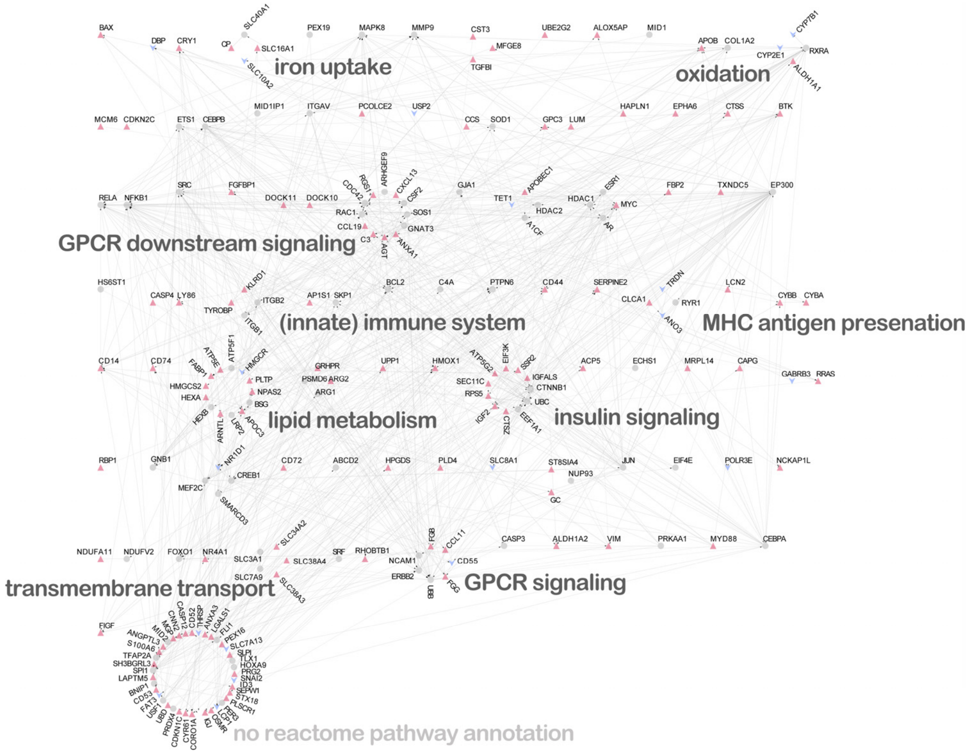

Figure S2. Reactome pathway enrichment of differentially expressed genes. Reactome FI was used to generate this network of enriched pathways, with one linker node allowed. Genes were annotated with and clustered based on Reactome pathways. In accordance to GO term enrichment, pathways that were enriched include lipid metabolism, G-protein-coupled receptor (GPCR) signaling, (innate) immune response, as well as iron metabolism. Up-regulated genes are represented by red triangles, down-regulation is indicated using blue down-arrows. Linker nodes are shown as grey circles.

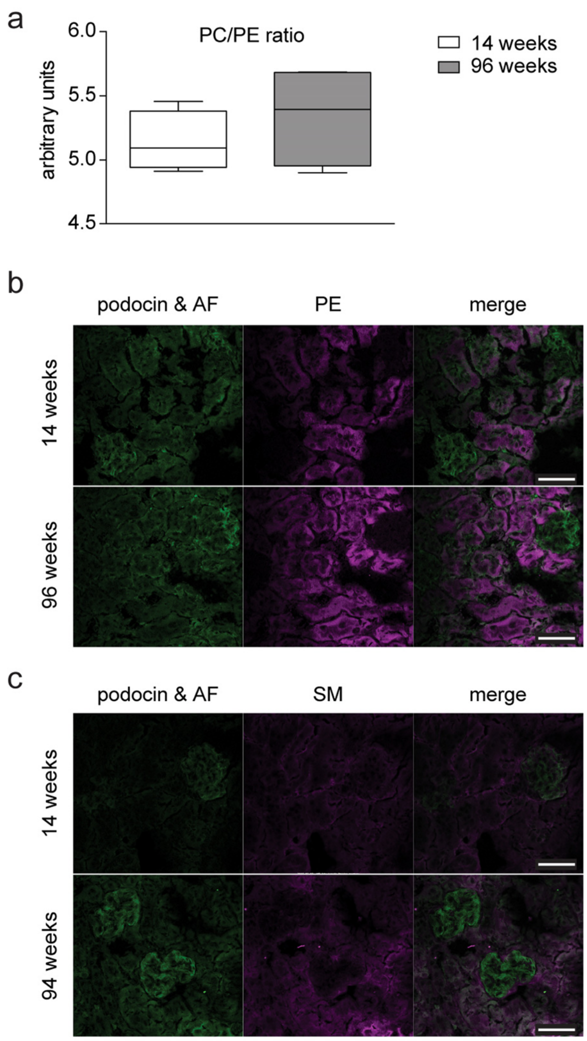

Figure S3. PC/PE ratio and lipid stainings show no difference between age groups. (a) Phosphatidylcholine/Phosphatidylethanolamine (PC/PE) ratio **(b)** PE staining on young and aged kidneys. Stainings show a high abundance in the tubular system without glomerular staining. **(c)** SM staining on young and aged kidneys. Little abundance can be detected in glomeruli. Tubuli show interstitial and intracellular staining. Podocin and autofluorescence (AF) were used as a control. Boxplots depict mean values with whiskers showing 5-95% percentile.

Supplemental Table S1. qPCR primer sequences and assay numbers

Gene	Primer	Sequence (5'-3')	Commer-cial	No.
**Fabp1**	Forward	CGAACTGGAGACCATGACTGG	No	
	Reverse	CAATGTCATGGTATTGGTGATTGTG	No	
**Kim1**	Forward	AAGAAATCTAGGTCAGTACCATGAA	No	
	Reverse	CACCACCCCCTTTACTTCCA	No	
**Lcn2**	Forward	CCCCATCTCTGCTCACTGTC	No	
	Reverse	TTTTTCTGGACCGCATTG	No	
**Aldh1a1**	probe based		Yes	Mm00657317_m1
**Atp4a**	probe based		Yes	Mm00444417_m1
**B2m**	probe based		Yes	Mm00437762_m1
**Cd74**	probe based		Yes	Mm00658576_m1
**H2-Aa**	probe based		Yes	Mm00439211_m1
**Lyz2**	probe based		Yes	Mm01612741_m1
**Vstm2a**	probe based		Yes	Mm00461305_m1
**Pigr**	probe based		Yes	Mm00465049_m1
**Slc7a13**	probe based		Yes	Mm00503856_m1
**Prlr**	probe based		Yes	Mm00599957_m1
**Npy6r**	probe based		Yes	Mm00627550_m1
